# Serum Extracellular Superoxide Dismutase Is Associated with Diabetic Retinopathy Stage in Chinese Patients with Type 2 Diabetes Mellitus

**DOI:** 10.1155/2018/8721379

**Published:** 2018-04-22

**Authors:** Jin-Song Zhao, Hui-Xiang Jin, Jia-Lin Gao, Chun Pu, Peng Zhang, Jian-Jun Huang, Long Cheng, Gang Feng

**Affiliations:** ^1^Department of Biochemistry, Wannan Medical College, Wuhu, Anhui 241001, China; ^2^Department of Ophthalmology, The First Affiliated Hospital of Wannan Medical College, Wuhu, Anhui 241001, China; ^3^Department of Endocrinology and Genetic Metabolism, The First Affiliated Hospital of Wannan Medical College, Wuhu, Anhui 241001, China; ^4^Clinical Laboratory, The First Affiliated Hospital of Wannan Medical College, Wuhu, Anhui 241001, China

## Abstract

Extracellular superoxide dismutase (ecSOD) is the major extracellular scavenger of reactive oxygen species and associated with the diabetic complication in patients with type 2 diabetes mellitus (T2DM). We aimed to investigate the serum ecSOD activity in Chinese patients with different stages of diabetic retinopathy (DR) and evaluate the association between the serum ecSOD activity and the severity of DR. A total of 343 T2DM patients were categorized into three groups: nondiabetic retinopathy (NDR) group, nonproliferative diabetic retinopathy (NPDR) group, and proliferative diabetic retinopathy (PDR) group. Serum ecSOD activities were measured by the autoxidation of the pyrogallol method. In this study, 271, 46, and 26 patients were enrolled in the NDR, NPDR, and PDR groups, respectively. We found a significantly decreased trend of serum ecSOD activity among NDR subjects (118.0 ± 11.5 U/mL) compared to NPDR subjects (108.5 ± 11.9 U/mL) (*P* < 0.001) and NPDR subjects compared to PDR subjects (102.7 ± 12.4 U/mL) (*P* = 0.041). Serum ecSOD activity was an independent risk factor for DR (OR = 0.920, *P* < 0.001) and was associated with the progression of DR. Serum ecSOD activity might be a biomarker for DR screening and evaluation of the clinical severity of DR in Chinese T2DM patients.

## 1. Introduction

Diabetic retinopathy (DR) is the most common and severe microvascular complication in patients with type 2 diabetes mellitus (T2DM), which is the main cause of blindness or serious visual impairment [1]. The cause of DR has not been fully elucidated; vascular endothelial cell injury or endothelial dysfunction may play important roles. Chronic uncontrolled hyperglycemia is thought to be the crucial influencing factor for the dysfunction of retinal microvascular pericyte and endothelial cell in T2DM patients [2]. Hyperglycemia can result in the overproduction of reactive oxygen species (ROS) such as hydroxyl and superoxide radicals. The accumulation of ROS can lead to oxidative stress, which is one of the key factors of the pathogenesis of DR [3]. Because of high energy demands and exposure to light, the retina is especially more susceptible to oxidative damage [4]. Various mechanisms contributing to the pathogenesis of DR such as inflammation, polyol pathway, accumulation of advanced glycation end products (AGEs), flux of hexosamine pathway, and protein kinase C (PKC) activation are associated with the overproduction of ROS by the mitochondria [5]. Maugeri et al. [6] have demonstrated that the pituitary adenylate cyclase-activating polypeptide (PACAP) and vasoactive intestinal peptide (VIP) could inhibit the hyperglycemia/hypoxia-induced retinal dysfunction in patients with DR. Therefore, the markers of oxidative stress were worthy of further study in patients with DR.

Superoxide dismutases (SOD) are the most important antioxidant defense systems against ROS, particularly catalyzing the dismutation of superoxide anion (O_2_^−^) into hydrogen peroxide and molecular oxygen [7]. According to localization in different cellular compartments, three major isoforms of SOD have been described. Cu-Zn-SOD is found in the cytoplasm, Mn-SOD localizes in the intermembrane space of the mitochondria or mitochondrial matrix, and extracellular SOD (ecSOD) is found in the extracellular matrix of tissues [8, 9]. The ecSOD is a secretory glycoprotein and bound to heparin sulfate on the cellular surface, accounting for more than 70% of total SOD activity in human vessels [10]. In the last years, various studies have reported the association between ecSOD and diabetic complication including albuminuria [11], nephropathy [12], cardiovascular disease [13], and diabetic foot ulcer [14].

However, until now, no study has investigated the association between ecSOD and DR, especially in Chinese T2DM patients. Thus, this study aimed to evaluate the association between serum ecSOD activity and DR severity in Chinese T2DM patients.

## 2. Methods and Subjects

### 2.1. Patient Eligibility

This was a hospital-based cross-sectional study which included the T2DM patients who were referred to the Ophthalmology Department of the First Affiliated Hospital of Wannan Medical College between May 2016 and April 2017 for DR screening. Patients were excluded if they met the following criteria: (1) having other severe diabetic complications, such as ketoacidosis, hyperosmotic coma, or nephropathy; (2) having other major systemic disorders or malignancy; (3) having other ocular diseases or a history of any type of intraocular surgery; and (4) having antioxidant therapy.

DR was diagnosed and classified by a single experienced ophthalmologist according to the results of indirect ophthalmoscopy. All selected patients were categorized into three groups: nondiabetic retinopathy (NDR) group, nonproliferative diabetic retinopathy (NPDR) group, and proliferative diabetic retinopathy (PDR) group. All participants were living in the same geographic area (Wuhu City, Anhui Province, China). Information on age, gender, duration of T2DM, systolic blood pressure (SBP), diastolic blood pressure (DBP), and body mass index (BMI) was obtained from all participants. This study was reviewed and approved by the Ethics Committee of Wannan Medical College, and signed informed consent was obtained from all the participants.

### 2.2. Sample Collection and Measurement

All venous blood specimens were collected under fasting conditions (overnight at least 8 h), delivered to the biochemistry laboratory of the First Affiliated Hospital of Wannan Medical College, and measured within 4 h. Serum ecSOD activity was measured using the autoxidation of the pyrogallol method (Superoxide dismutase Assay Kit, Fuyuan Biotechnology Co. Ltd., Fujian, China), following the manufacturer's instructions. Glycated hemoglobin (HbA1c) was performed by high-performance liquid chromatography (Variant II; Bio-Rad Laboratories, Hercules, CA, USA). Fasting serum glucose (FBG), serum creatinine (Cr), serum blood urea nitrogen (BUN), serum cystatin c (CYS-C), serum triglycerides (TG), serum total cholesterol (TC), serum low-density lipoprotein cholesterol (LDL-C), and serum high-density lipoprotein cholesterol (HDL-C) were measured using the standard techniques by an automatic analyzer (Hitachi 7060; Hitachi High Technologies, Tokyo, Japan).

### 2.3. Statistical Analysis

Continuous variables were normally distributed by the Kolmogorov–Smirnov test and provided as the mean ± SD. Categorical variables were expressed as percentages. Overall comparisons were performed with one-way ANOVA, and multiple comparisons between the two groups were derived from the LSD *t*-test. Differences in percentages of variables were determined by a chi-squared test. The univariate correlations between serum ecSOD activity and other variables were determined by the Pearson correlation analysis. Afterward, multivariate linear regression analysis was performed to determine the independent relationships. A multivariate logistic regression model was constructed to predict DR in T2DM patients. A nomogram was formulated based on the results of multivariate logistic regression analysis and by using R version 3.3.2 (http://www.r-project.org/). During the external validation of the nomogram, the total points of each patient in the validation cohort were calculated according to the established nomogram. The receiver operating characteristic (ROC) curve and the corresponding area under the curve (AUC) were calculated for testing the potential of serum ecSOD activity for the identification of DR, NPDR, and PDR in T2DM patients. All statistical analyses were performed using the SPSS 19.0 version (SPSS Inc., Chicago, IL, USA) and R version 3.3.2. A two-sided *P* value < 0.05 was considered statistically significant.

## 3. Results

A total of 343 T2DM patients were enlisted in the study, including 159 females (46.4%) and 184 males (53.6%); the mean age was 57.7 ± 11.7 years (range 28–85). All of the participants were self-identified as Han Chinese. Among these patients, 271 patients (79.0%) were included in the NDR group, 46 patients (13.4%) were included in the NPDR group, and 26 patients (7.6%) were included in the PDR group. No difference was observed in gender (*P* = 0.219) between T2DM patients with DR and without DR. Compared with T2DM patients without DR, T2DM patients with DR had higher age, longer duration of T2DM, higher SBP, higher FBG, higher HbA1c, and higher CYS-C. All characteristics of the subjects are summarized in Table 1.

**Figure 1:**
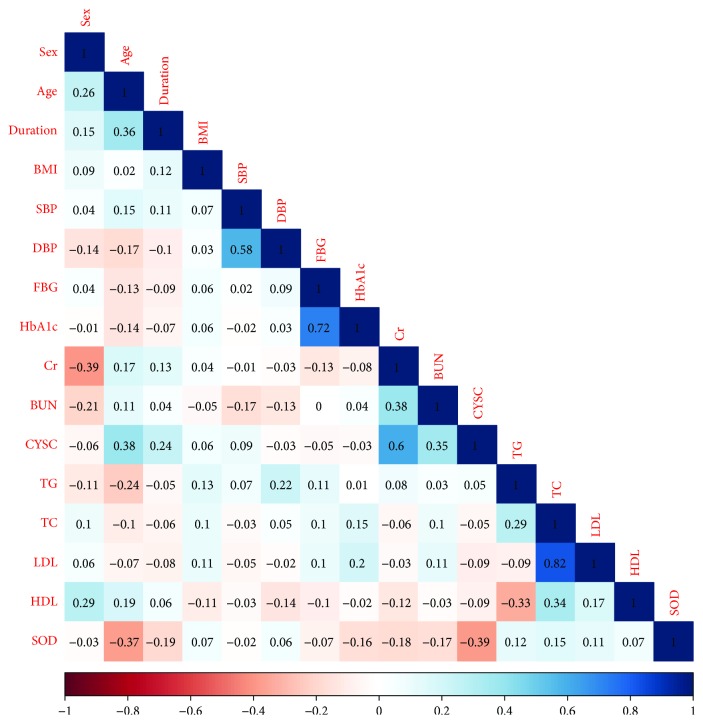
Univariate correlations of all variables in T2DM patients.

By the Pearson correlation analysis, serum ecSOD activity was significantly correlated with CYS-C (*r* = −0.39, *P* < 0.001), age (*r* = −0.37, *P* < 0.001), duration of T2DM (*r* = −0.19, *P* = 0.001), Cr (*r* = −0.18, *P* = 0.001), BUN (*r* = −0.17, *P* = 0.002), HbA1c (*r* = −0.16, *P* = 0.002), TC (*r* = 0.15, *P* = 0.005), TG (*r* = 0.12, *P* = 0.027), and LDL-C (*r* = 0.11, *P* = 0.038) (Figure 1). Data (data1.csv) and R codes of Figure 1 are given in Supplemental Materials (available here). Multivariate linear regression analysis between serum ecSOD activity (dependent variable) and clinical characteristics (independent variables) showed that age (*β* = −0.326, *P* < 0.001), HbA1c (*β* = −1.532, *P* < 0.001), and CYS-C (*β* = −10.561, *P* < 0.001) were the independently negative factors for serum ecSOD activity, whereas HDL-C (*β* = 8.121, *P* = 0.012) was the independently positive factor (Table 2).

**Figure 2:**
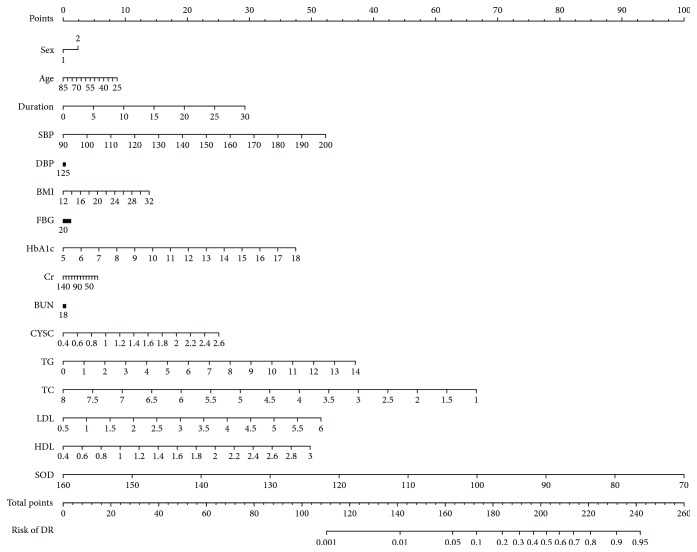
Risk factors of DR nomogram (code of sex [1: male, 2: female]). (To use the nomogram, an individual patient's value is located on each variable axis, and a line is drawn upward to determine the number of points received for each variable value. The sum of these numbers is located on the total points' axis, and a line is drawn downward to the risk of DR's axes to determine DR risk.)

**Figure 3:**
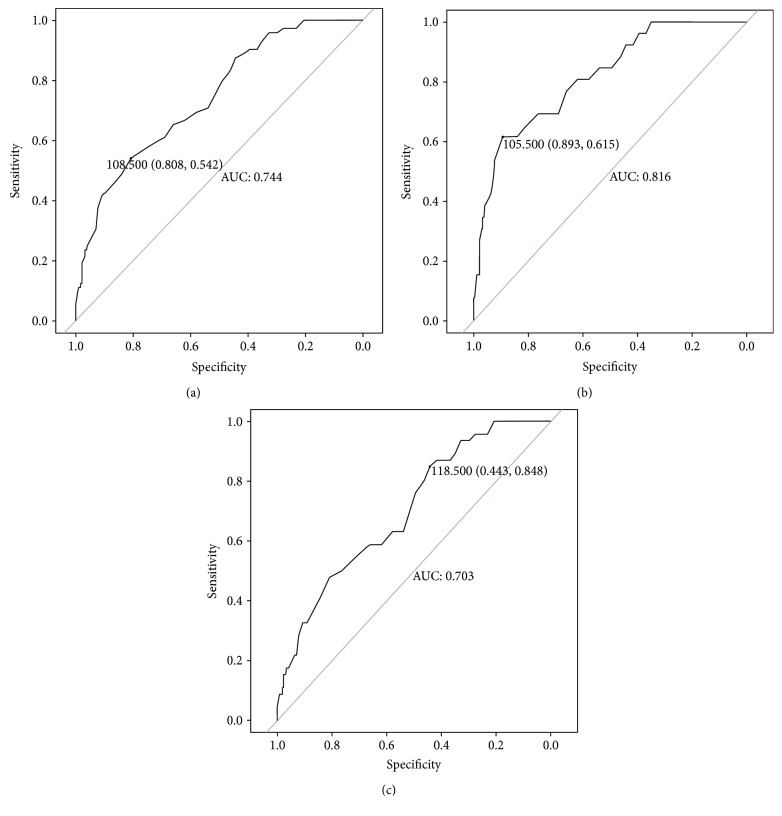
Receiver operating characteristic (ROC) curve of serum ecSOD activity in identification of T2DM patients with DR (a), NPDR (b), and PDR (c).

Compared with the NDR group (118.0 ± 11.5 U/mL), the serum ecSOD activity was significantly decreased in the DR group (106.4 ± 12.3 U/mL, *P* < 0.001). By multivariate logistic regression analysis, duration of T2DM (OR = 1.076, *P* = 0.004), SBP (OR = 1.029, *P* = 0.017), HbA1c (OR = 1.241, *P* = 0.024), and serum ecSOD activity (OR = 0.920, *P* < 0.001) were the independent and significant risk factors of DR in T2DM patients (Figure 2). Data (data2.csv) and R codes of Figure 2 are given in Supplemental Materials. As shown by the ROC curve (Figure 3(a)), DR could be identified with a sensitivity of 54.2% at a specificity of 80.8% (AUC = 0.744, 95% CI 0.682–0.806) by serum ecSOD activity (cutoff value = 108.5 U/mL) in our study population.

**Figure 4:**
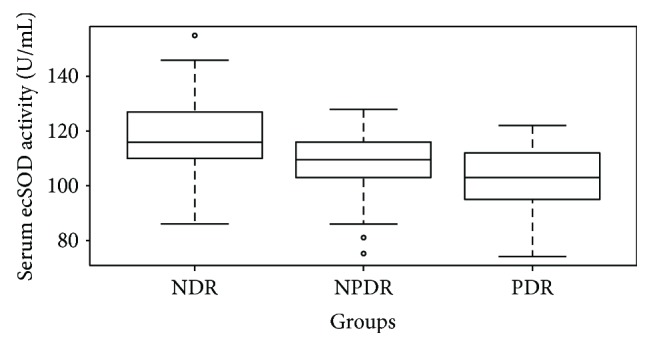
Serum ecSOD activity (mean ± SD) in the nondiabetic retinopathy (NDR) group, nonproliferative diabetic retinopathy (NPDR) group, and proliferative diabetic retinopathy (PDR) group.

Figure 4 shows a significantly decreased trend of serum ecSOD activity among NDR subjects (118.0 ± 11.5 U/mL) compared to NPDR subjects (108.5 ± 11.9 U/mL) (*P* < 0.001) and NPDR subjects compared to PDR subjects (102.7 ± 12.4 U/mL) (*P* = 0.041). NPDR could be identified with a sensitivity of 84.8% at a specificity of 44.3% (AUC = 0.703, 95% CI 0.626–0.781) by serum ecSOD activity (cutoff value = 118.5 U/mL) (Figure 3(b)), and PDR could be identified with a sensitivity of 61.5% at a specificity of 89.3% (AUC = 0.816, 95% CI 0.733–0.900) by serum ecSOD activity (cutoff value = 105.5 U/mL) (Figure 3(c)).

## 4. Discussion

In this study, a high level of HbA1c and FPG had been found in all groups, with the DR group having a greater increase as a consequence of poorly controlled glycemia in these subjects. Although the mechanisms of how increased glucose levels contribute to DR have not been fully elucidated, hyperglycemia-induced oxidative stress is considered an important cause. The present study investigated the possible relationship between serum ecSOD activity and other variables in Chinese T2DM patients. The data indicated that serum ecSOD activity was negatively correlated with age, duration of T2DM, HbA1c, CYS-C, Cr, and BUN, thus suggesting that the decreased serum ecSOD activity was associated with poor glycemic control and the development of microvascular damage. We observed a positive correlation between serum ecSOD activity and TG, TC, and LDL-C, which is in agreement with the previous report by Gómez-Marcos et al. [15]. However, further studies are required to elucidate the mechanism behind their associations.

Controversial reports on changes in serum/plasma ecSOD activity of T2DM patients have been published. In some, a decrease in the activity was observed [16], whereas in others an increase was reported [17, 18]. In this study, a significant decrease in serum ecSOD activity in T2DM patients and a substantial reduction in the DR group were observed, according to the reference range of serum ecSOD activity (129–216 U/mL) in our laboratory. In a previous literature [19], the glycation of the enzyme had been reported to occur frequently in diabetes with poor glycemic control. The high level of HbA1c and FPG had been found in this study population, especially in the PDR group. Thus, one possible consideration is that the glycation decreases the serum ecSOD activity.

Conventional risk factors of DR such as diabetes duration, HbA1c, and SBP have been found in our population by multivariate logistic regression analysis [20, 21]. Furthermore, we observed that serum ecSOD activity was also an independent risk factor of DR. DR could be identified with a sensitivity of 54.2% at a specificity of 80.8% (AUC = 0.744) by serum ecSOD activity (cutoff value = 108.5 U/mL) in our study population. Our finding suggests that serum ecSOD activity might be a biomarker for DR screening in T2DM patients.

The gold standard for diagnosing different stages of DR is fundus fluorescence angiography (FFA) [22]. However, FFA is an invasive procedure and causes discomfort to patients, which is hard to use in clinical experiences generally. Furthermore, some districts suffer from weak health infrastructure, in terms both of medical equipment and of professional technical staff, which cannot carry out FFA. In the present study, we observed the serum ecSOD activity reduced gradually as NDR progressed to NPDR and NPDR progressed to PDR. This decreased trend indicates that serum ecSOD activity is associated with the progression of DR. Based on the above research, two special diagnostic thresholds could be determined. When serum ecSOD activity was lower than 118.5 U/mL, there was an increased risk of developing NPDR. When serum ecSOD activity was lower than 105.5 U/mL, it is more likely to be PDR. Furthermore, when serum ecSOD activity is within the range of 105.5–118.5 U/mL, we consider that the endothelial cells are injured most severely and NPDR rapidly develop into PDR at this stage. Thus, early interventions of DR are urgently needed for the patients in this key stage. Compared with FFA, the measurement of serum ecSOD activity was less invasive and could be used routinely in clinical laboratories. If our results are confirmed by other groups, this marker can easily be implemented into clinical application for DR screening and the evaluation of the clinical severity of DR.

This study had some limitations. First, DR was classified using indirect ophthalmoscopy rather than FFA. The DR degree might have been underestimated in some patients [23]. Second, all study subjects were recruited from a single hospital. Whether our findings can be extended to the general population remains to be determined. Third, we have not investigated AGEs and some other markers of oxidative stress, such as glutathione peroxidase and catalase [24–26]. Furthermore, some factors of DR promotion including homocysteine were not been investigated in our study [27]. Finally, our results were from the cross-sectional study and could not prove causality but only demonstrate associations. Prospective or longitudinal studies of large samples are clearly required to validate an association between the serum ecSOD activity and the progression of DR and to validate whether such an association could help identify subjects at a high risk of developing DR.

## 5. Conclusions

In conclusion, we observed that serum ecSOD activity reduced gradually with the increase of DR degree. Serum ecSOD activity might be a biomarker for DR screening and evaluation of the clinical severity of DR in Chinese T2DM patients.

## Figures and Tables

**Table 1:** The characteristics of subjects (*n* = 343).

Characteristics	NDR group (*n* = 271)	DR group (*n* = 72)	NPDR group (*n* = 46)	PDR group (*n* = 26)
Age (years)	56.5 ± 11.6	62.0 ± 10.8^∗^	60.9 ± 9.7^∗^	63.9 ± 12.6^∗^
Gender (F/M)	121/150	38/34	22/24	16/10
Duration of T2DM (years)	6.8 ± 5.8	10.7 ± 7.4^∗^	10.9 ± 7.4^∗^	10.4 ± 7.5^∗^
BMI (kg/m^2^)	22.3 ± 3.3	22.8 ± 3.3	22.7 ± 3.4	22.9 ± 3.3
SBP (mmHg)	131.5 ± 16.1	138.6 ± 17.4^∗^	136.1 ± 14.8	143.0 ± 20.9
DBP (mmHg)	82.6 ± 10.6	83.8 ± 9.9	83.1 ± 9.5	85.0 ± 10.6
FBG (mmol/L)	8.59 ± 3.45	9.66 ± 3.90^∗^	9.38 ± 4.11	10.16 ± 3.51^∗^
HbA1c (%)	8.8 ± 2.2	9.9 ± 2.4^∗^	9.7 ± 2.4^∗^	10.2 ± 2.3^∗^
Cr (*μ*mol/L)	67.8 ± 18.3	72.9 ± 26.7	70.8 ± 26.2	76.5 ± 27.4^∗^
BUN (mmol/L)	5.63 ± 1.97	5.88 ± 1.86	5.82 ± 1.91	6.00 ± 1.78
CYS-C (mg/L)	1.01 ± 0.28	1.20 ± 0.41^∗^	1.17 ± 0.40^∗^	1.27 ± 0.42^∗^
TG (mmol/L)	1.78 ± 1.45	1.67 ± 1.67	1.65 ± 1.61	1.70 ± 1.79
TC (mmol/L)	4.07 ± 1.05	3.88 ± 0.89	3.83 ± 0.89	3.96 ± 0.90
LDL-C (mmol/L)	2.22 ± 0.79	2.11 ± 0.67	2.07 ± 0.63	2.17 ± 0.73
HDL-C (mmol/L)	1.21 ± 0.32	1.21 ± 0.27	1.21 ± 0.27	1.20 ± 0.28

$\begin{array}{c} {}^{*}P<0.05 \end{array}$ versus NDR.

**Table 2:** Multivariate linear regression analysis between serum ecSOD activity (dependent variable) and clinical characteristics (independent variables).

	*β* (95% confidence interval)	SE	*P* value
Age	−0.326 (−0.450 to −0.201)	0.063	<0.001
Gender	−0.110 (−2.972 to 2.752)	1.455	0.940
Duration of T2DM	−0.097 (−0.294 to 0.101)	0.100	0.336
BMI	0.365 (0.01 to 0.728)	0.185	0.051
SBP	0.052 (−0.039 to 0.143)	0.046	0.259
DBP	−0.053 (−0.199 to 0.093)	0.074	0.478
FBG	0.237 (−0.241 to 0.715)	0.243	0.330
HbA1c	−1.532 (−2.270 to −0.795)	0.375	<0.001
Cr	0.029 (−0.054 to 0.111)	0.042	0.495
BUN	−0.259 (−0.935 to 0.417)	0.344	0.452
CYS-C	−10.561 (−15.675 to −5.447)	2.600	<0.001
TG	1.477 (−0.055 to 3.010)	0.779	0.059
TC	−2.042 (−6.011 to 1.927)	2.017	0.312
LDL-C	3.730 (−0.814 to 8.274)	2.310	0.107
HDL-C	8.121 (1.822 to 14.419)	3.202	0.012

## Supplementary Materials

Supplementary Materials Data 1. Data 2. R codes.
