# Supplementary material for: Serum Extracellular Superoxide Dismutase Is Associated with Diabetic Retinopathy Stage in Chinese Patients with Type 2 Diabetes Mellitus
Source: Dis Markers. 2018 Apr 22;2018:8721379. doi: 10.1155/2018/8721379 (PMC5937587; doi:10.1155/2018/8721379)
Supplement: Supplementary Materials — Data 1. Data 2. R codes. [file 8721379.f1.zip › R codes.doc]

##Univariate correlations

P<- read.csv("D:/R/data1.csv")

attach(P)

require(corrplot)

corrplot(corr =cor(P), method = "color", type="lower", order = "original", addCoef.col = "black")

##Multivariate logistic regression analysis

P<- read.csv("D:/R/data2.csv")

attach(P)

require(rms)

dd <- datadist(P)

options(datadist="dd")

f <- lrm(DIA ~SEX+AGE+DURATION+SBP+DBP+BMI+FBG+HbA1c+Cr+BUN+CYSC+TG+TC+LDL+HDL+SOD)

nom <- nomogram(f, fun=plogis, fun.at=c(.001, .01, .05, seq(.1,.9, by=.1), .95, .99, .999), lp=F, funlabel="Risk of DR")

plot(nom, xfrac=.1)
